# Field pest monitoring and forecasting system for pest control

**DOI:** 10.3389/fpls.2022.990965

**Published:** 2022-08-29

**Authors:** Chengkang Liu, Zhiqiang Zhai, Ruoyu Zhang, Jingya Bai, Mengyun Zhang

**Affiliations:** ^1^College of Mechanical and Electrical Engineering, Shihezi University, Shihezi, China; ^2^Key Laboratory of Northwest Agricultural Equipment, Ministry of Agriculture and Rural Affairs, Shihezi, China

**Keywords:** cotton pest, deep learning, image acquisition device, insect outbreak, transfer learning

## Abstract

Insect pest is an essential factor affecting crop yield, and the effect of pest control depends on the timeliness and accuracy of pest forecasting. The traditional method forecasts pest outbreaks by manually observing (capturing), identifying, and counting insects, which is very time-consuming and laborious. Therefore, developing a method that can more timely and accurately identify insects and obtain insect information. This study designed an image acquisition device that can quickly collect real-time photos of phototactic insects. A pest identification model was established based on a deep learning algorithm. In addition, a model update strategy and a pest outbreak warning method based on the identification results were proposed. Insect images were processed to establish the identification model by removing the background; a laboratory image collection test verified the feasibility. The results showed that the proportion of images with the background completely removed was 90.2%. Dataset 1 was obtained using reared target insects, and the identification accuracy of the ResNet V2 model on the test set was 96%. Furthermore, Dataset 2 was obtained in the cotton field using a designed field device. In exploring the model update strategy, firstly, the T_ResNet V2 model was trained with Dataset 2 using transfer learning based on the ResNet V2 model; its identification accuracy on the test set was 84.6%. Secondly, after reasonably mixing the indoor and field datasets, the SM_ResNet V2 model had an identification accuracy of 85.7%. The cotton pest image acquisition, transmission, and automatic identification system provide a good tool for accurately forecasting pest outbreaks in cotton fields.

## Introduction

Pest forecasting refers to the accurate monitoring of pests and predicting pest outbreaks through analyzing relevant data based on biology, mathematics, statistics, etc. It is very important for farmers to achieve good pest control. Accurate and timely pest forecasting can help farmers timely take measures to effectively control agricultural pests and reduce the detrimental effects of pesticide abuse on the environment and the human body (Xiao et al., 2000; Dalal and Singh, 2017; Malschi et al., 2018; Hu et al., 2019; Machekano et al., 2019; Ponomarev et al., 2019).

The traditional monitoring methods obtain pest information by regular field surveys (Ebrahimi et al., 2017). Although field surveys can obtain detailed pest information, it is time-consuming, labor-intensive, and limited in large scale surveys (Dong et al., 2019; Li et al., 2019). Therefore, some studies perform pest monitoring using sticky boards combined with wifi cameras to collect pest information, and others perform pest monitoring using white screens with specific light sources to trap specific insects. Although these two methods can obtain good results, they have obvious drawbacks when compared with the predictive model, such as regular replacements of sticky boards, lacks unified color expression pattern for the sticky color palette, and limited target insect range (Kumar et al., 2010; Zou et al., 2014; Chen et al., 2016; Chiu et al., 2019). In recent years, geographic information systems (GIS) have been used in agricultural pest studies. A GIS is a computer system that collects, stores, and analyses data about the entire or a portion of the Earth’s surface and displays geographic distribution. Various functions are performed during the entire process of data collection, processing, and decision-making (Zhang, 2000). The obtained data could be evaluated in depth in both time and space dimensions using GIS technology paired with insect pest monitoring and early warning models, thus achieving accurate monitoring and early warning services. However, personnel are still necessary for the data collection in the field and the operation is very time-consuming (Xu et al., 2017; Dong et al., 2019; Afonin et al., 2020). Therefore, a system that could collect real-time insect data in a fast and accurate manner is needed for pest control.

Pest identification and classification are crucial for pest forecasting. Automatic pest detection has become a research topic in recent years with the rapid development of machine learning and machine vision (Li et al., 2019). Image segmentation and other approaches have been used to preprocess the images, after which the features of target images are extracted. Then, machine learning methods, such as random forest, k nearest neighbors, support vector machine (SVM), etc. are used to perform pest identification (Rajan et al., 2016; Yuan and Hu, 2016; Ebrahimi et al., 2017; Javed et al., 2017; Zhang et al., 2019). Although the approaches described above have produced good identification results, they have flaws such as insufficient extracted information, manual identification of critical features, and low feature extraction efficiency. Deep convolutional neural networks (DCNNs) have become an effective method to solve the problems mentioned above in recent years (Liu et al., 2016). Furthermore, deep learning technology based on convolutional neural networks (CNNs) has made significant progress in identifying objects, such as pests. Due to the poor identification performance of CNNs in the field, Li et al. (2019) proposed a data augmentation approach based on CNNs. Liu et al. (2016) proposed a method based on a saliency map and applied it to DCNNs for pest identification in a rice field, and found that the accuracy was better than that of previous. Nam and Hung (2018) successfully employed CNN to classify and identify harmful bugs in traps. Alfarisy et al. (2018) created a rice pest identification model using the Caffe framework. To evaluate the model’s prediction accuracy, Thenmozhi and Reddy (2019) compared their constructed CNN model with several migration models based on multiple deep learning architectures. Chen et al. (2021) proposed an interpretable CNN model to visualize and interpret pest identification results. Singh et al. (2021) compared multiple CNN pre-trained models and deployed the optimal model in a web application for automatic disease detection in coconut trees. Nanni et al. (2022) trained on the Deng (SMALL), large IP102, and Xie2 (D0) pest datasets based on a collection of CNNs with different structures and found that the models obtained using different combinations of CNNs with different Adam performed best. Besides, to save time and resources on training; Khan and Ullah (2022) compared two CNN migration learning models on the IP102 dataset. The results show that the applied model outperforms existing classification algorithms on large data sets. Li et al. (2022) also used the IP102 dataset and compared the classification accuracy under different combinations of learning rate, migration learning and data enhancement methods. They found that the classification of models with transfer learning outperformed those based on new learning models, and that appropriate data enhancement techniques could improve classification accuracy (Li et al., 2022). Several researchers adopted transfer learning methods to carry out pest identification and classification and obtained excellent results (Dawei et al., 2019; Swasono et al., 2019; Chen et al., 2020; Khalifa et al., 2020; Pattnaik et al., 2020).

Based on the above mentioned successful application of CNNs in pest identification, it is vital to investigate the effects of employing CNNs in phototactic insect and moth identification systems. Therefore, this study tried to create a system for efficiently acquiring and identifying phototactic insect images. The objectives were to (1) design an image acquisition device; (2) collect datasets for pest identification; (3) train models for identifying three target pests in cotton fields; (4) provide an early warning method; and (5) investigate possible strategies for improving the model.

## Materials and methods

### Image acquisition device

#### Test device

##### Design of image acquisition platform

**FIGURE 1:**
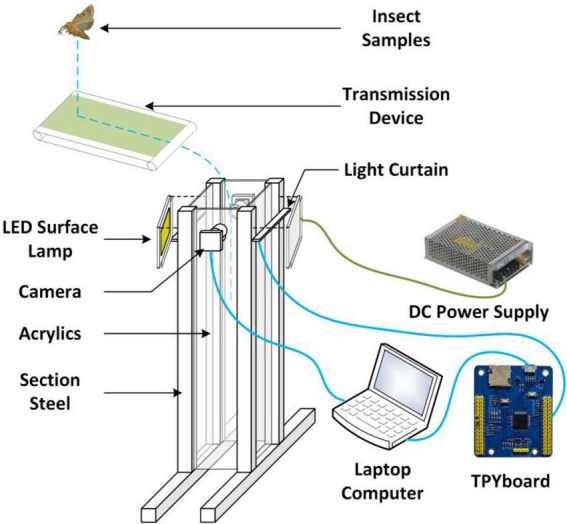
Insect image acquisition device.

The indoor insect image acquisition platform consisted of an image acquisition system, an insect body transmission system, sensors, light sources, and image acquisition software. The image acquisition system was the most important part, and others were integrated into the image acquisition system (Figure 1). Aluminum profiles were used to construct the bracket of the image acquisition system and the insect body transmission system. The length and width of the square mouth of the platform was 100 mm, and the height of the acquisition channel was 600 mm. The upper surface of the light curtain sensor was 200 mm away from the conveyer belt of the insect body transmission system, and the vertical distance between the axis of the driven shaft and the center of the camera was 100 mm. This could make the insects enter the falling passage in a parabola. Furthermore, two cameras (2.8–12 zoom, 1080p, Green Vision Forest, China) were positioned opposite each other to take two separate photos of the same insect body, and the center of the cameras was flush with the center of the light curtain sensor. Two light-emitting diode (LED) surface light sources were positioned on both sides of the sensor in parallel and at a 15-degree angle to the center line of the two cameras to ensure uniform lighting.

##### Sensor unit

The sensor unit was composed of two complementary metal oxide semiconductor (CMOS) cameras (2.8–12 zoom, 1080p, Green Vision Forest, China), a light curtain sensor, and a laptop computer. The cameras were connected to the computer *via* a USB port, and the signal line of the light curtain sensor (PT1000QL radiation sensor, switch sensor, BOJKE, China) was connected with the TPYboard (V102, MCU: stm32f405rgt6. The power supply line was connected to the power supply module (3 V/5 V/12 V multiple outputs, Xintai Microelectronics, Shenzhen, China), and the TPYboard was connected to the computer by USB. The computer has an Intel i5–4210 M processor with 8 GB of RAM and runs on Windows 10.

##### Data acquisition

**FIGURE 2:**
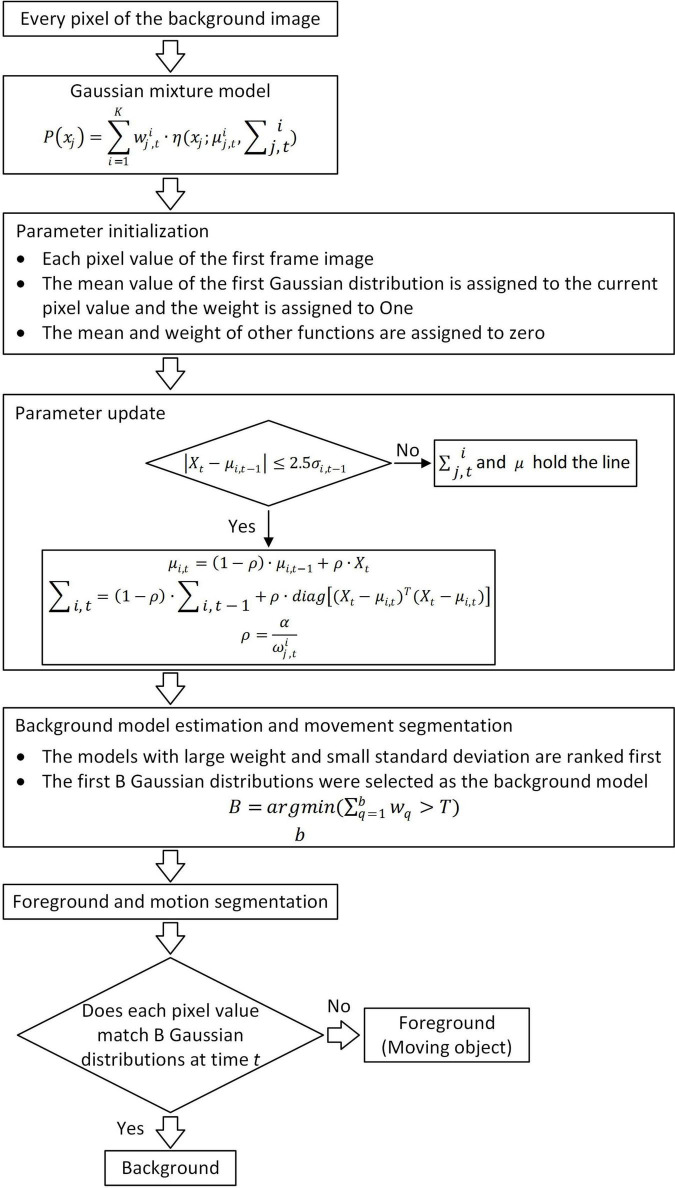
Flowchart of the background subtraction algorithm.

An image acquisition application is written using Python, and the computer received signals from the light curtain sensor and controlled the cameras to collect insect images. Image background removal was performed during data acquisition using the Gaussian mixture model (Figure 2; Li and Zhao, 2011) to aid in the classification and identification of insects in the images. Gaussian mixture model is a method that locates the background through the background model and extracts the foreground from the image (Li and Zhao, 2011). The selection of the background model is vital. The background model, for example, works by first defining numerous Gaussian models, initializing parameters, solving the important parameters that will be utilized later, and then processing each pixel in each image frame so that it is visible. If it matches a previously defined Gaussian model, the pixel value is included in the model, and the model is updated to reflect the new pixel value. If it does not match, a new Gaussian model with the pixel value will be created, and its parameters are set to replace the most unlikely background model in the original model. Finally, the first few most likely models are selected as the background models.

The acquisition signal was sent from the light curtain sensor to the data acquisition application through the TPYboard. The data collection application activated two cameras to complete the image acquisition, and both photos with and without background removal were saved.

##### Feasibility analysis

To evaluate the image acquisition performance of the device, a statistical analysis of the acquired insect images was performed. The collected insect images were classified into six categories, including (a) images with no background and no noises, (b) images with removable noises, (c) images with non-removable noises, (d) images outside the camera’s field of view, (e) images in which the background was not removable, and (f) images in which the targets were blurred. The feasibility of the image acquisition approach was verified by analyzing the proportions of various categories of images.

#### Field device

##### Design of image acquisition device

**FIGURE 3:**
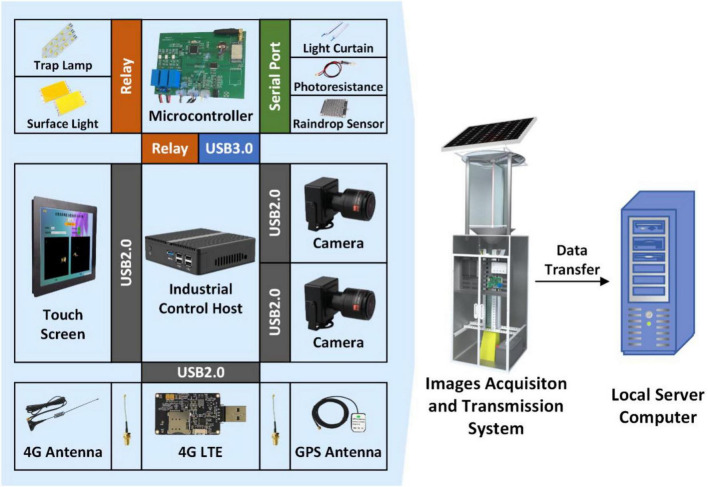
Image acquisition system.

The device was composed of a machine frame, insect stun system, sensor, electric control system, insect body collection box, and image acquisition and transmission software (Figure 3). To complete the acquisition and transmission of insect images, various sections were merged. A solar panel was mounted and the electric energy was stored in a battery on the machine’s bracket to power the device. Under the solar panel, an insect enticing and stunning system employed a full-band light source to attract field phototactic insects, and three transparent screens with a 120-degree angle were mounted surrounding the light source to achieve insect impact and stunning. When the insect was stunned, it fell into the lower funnel, which was connected to the upper part of the image acquisition system. The device’s image acquisition process was similar to that of the device in section “Test device.” In front of the image acquisition system, an electric control cabinet with an industrial computer, a control panel, a charging controller, and a touch display was mounted. For capturing insects, there was an insect collection box beneath the image acquisition system. If rain was detected, a raindrop sensor mounted on the top of the machine’s bracket would power off the device. The photosensitive sensor was mounted on the device casing’s exterior controlled the external light signal.

##### Sensor system

The sensor system was composed of a collection device, on-off switch, and a transmission and positioning unit (EC20 USB DONGLE, COWITOP, Shenzhen, China). The acquisition and transmission unit was composed of a light curtain sensor and two CMOS cameras. The light curtain sensor (PT1000QL radiation sensor, switch sensor, BOJKE, China) was connected to the control board (stm32, Shenzhen, China). The two cameras (2.8–12 zoom, 1080p, Green Vision Forest, China) and the transmission and positioning unit (EC20 USB DONGLE, COWITOP, Shenzhen, China) were connected to the industrial computer *via* a USB cable. The transmission and positioning unit employed a 4G communication network and has GPS functionality. The industrial computer was composed of Intel i3–4010 CPU, 4 GB RAM, and Windows 7 operating system. The on-off power switch included a raindrop sensor unit and a photosensitive sensor unit (FZ-GG, photoresistor, Feizhi Electronic Technology Co., Ltd., China).

##### Power system

In the field, batteries (12V65Ah/10HR, HW-HSE-65-12, Hangzhou, China) powered the device, and the solar panels (PM: 80 W, VOC: 17.5 V, IM: 4.57A, China) charged the batteries. The entire device started when the light was less than the threshold of the photosensitive sensor unit in the evening, and the raindrop sensor unit did not receive a signal. The control board regulates the power relay for the insect trap lamp (DC12 V 20 W 500 mm × 20 mm, Hangzhou Zhuoqi, China), touch screen (RG-XSQ008, HDMI VGA DC, 12-inch LED touch screen, Rongge, China), LED surface light source (DC12 V 20 W 94 mm × 50 mm, XEN-9450, Xing Yuan Sheng Optoelectronics, China), light curtain sensor, and the industrial computer. The trap lamp, industrial computer, and light curtain sensor were all turned on after the device was started. When the light curtain sensor detected an insect body, the LED surface light source was turned on for 5 s to supplement the light and facilitate the image acquisition.

##### Data acquisition software

The image acquisition and transmission program was written using Python 3.6 and PyQt5. The industrial computer was set to launch the data acquisition software. After powering up the industrial computer, the program collected the device’s position information *via* the transmission and positioning unit. The program continuously monitored the signal sent by the sensor system, and the LED surface light source was turned on as the image acquisition light source when the light curtain sensor received a trigger signal. Simultaneously, the program commanded the cameras to acquire the images (JSON), which contains metadata including the name, location, and time. The data were stored in the allocated folder before transfering to the computer. Furthermore, when the program received shutdown signal from the raindrop sensor unit or photosensitive sensor unit, the program executed the shutdown command to power off the industrial computer.

The device number, position, images, image acquisition status, and image transmission status were all displayed on the program interface. To check the working status of the program, the program determined whether the acquisition status was normal by detecting the serial port of the camera, the serial port of the control panel, and the transmission connection and network status. The status of each were displayed on the interface at the same time.

### Sample preparation

#### Indoor acquisition of sample images

**FIGURE 4:**
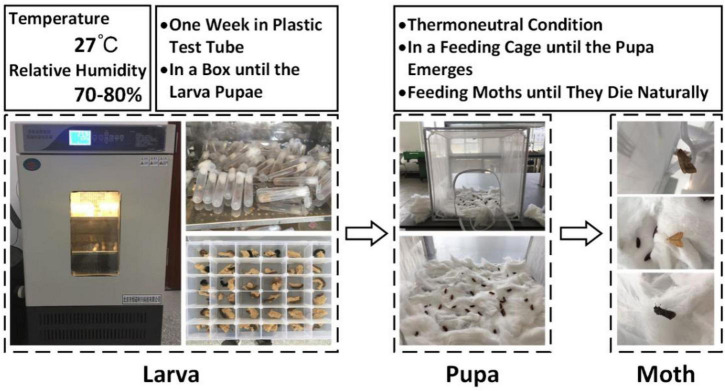
Insect rearing process.

Twenty-three instar larvae of cotton bollworm moth, borer moth, and Spodoptera litura were purchased and used as samples (Keyun Biology, Henan, China). In the larval stage, insects were reared in an incubator (SPX-50 L, Hongnuo, Hebei, China) at 27°C with a relative humidity of 70–80%. The larvae were kept in a pitcher at room temperature once they pupated (Figure 4). Sugar water was given to the pupae until they emerged. Cotton bollworm moths, borers, and prodenia litura had 20, 4, and 4 survivals, respectively, in the end. Ten cotton bollworm moths were selected as the samples, and six borers and six Spodoptera litura with intact bodies selected from the insects previously collected from the test field were included in the test samples to maintain consistency in the sample size.

#### Sample collection in cotton field

The cotton field (44°18′ 57′′ N and 86°03′ 61′′E) is located in Erlian, Shihezi City, Xinjiang Uygur Autonomous Region, China. During the flowering period of cotton, the device was placed on the border of the field to avoid interfering from the sprayer. On August 3, 2019, the device was deployed in the field and recovered on October 13, 2019.

### Image acquisition

**FIGURE 5:**
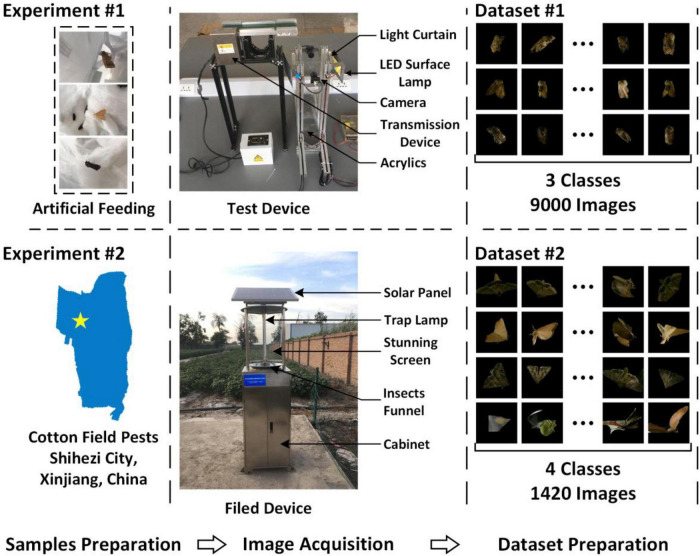
Data collection.

The first step was to acquire images using the device illustrated in section “Test device,” which was designed by the Intelligent Agricultural Sensing and Equipment Laboratory of Shihezi University. The pests were dropped at the calibrated position of the conveyor belt during image acquisition, and then they were transferred to the channel. A very short exposure time (312 μs) was set to assure the image quality for the cameras to acquire a higher frame rate (30 fps) due to the quick falling. Two 12 V 20 W LED surface light sources were used at the same time (Figure 5). There were three different categories of samples, each had 10 samples. Each sample had 400 images, and a threshold segmentation was used to select 300 images with no background or noises. Finally, 9,000 images for the three categories were obtained as Dataset 1. Then, Dataset 1 was divided into training, validation, and test sets according to a ratio of 7:1.5:1.5.

The image acquisition device in section “Image acquisition device,” which was collaboratively designed by the Intelligent Agricultural Sensing and Equipment Laboratory of Shihezi University and Hangzhou Zhuoqi Electronic Technology Co., Ltd., was used in the second step (Figure 5). A 15-acre (10,000-square-meter) trapping area was set aside. The device collected 111,146 images from August 3, 2019 to October 13, 2019. Between August 3 and August 10, insect experts of Shihezi University’s Agricultural College were invited to identify 80 cotton bollworm moths, 60 borer moths, and 80 Spodoptera litura images. In addition, 1200 images with non-target insects, distortion, and noises were considered as the fourth category. A total of 1,420 images were finally obtained for the four categories and defined as Dataset 2. Due to the minimal number of target insects identified manually, Dataset 2 was divided into training, validation, and test sets at a ratio of 6:2:2.

### Image classification

#### Image pre-processing

The images were cropped to obtain the region of interest (ROI), and the unnecessary information in the images was removed. The centroid of the insect body was used as the center of the rectangular box when cropping, and the ROI (140 × 140 pixel) was intercepted. The image’s border was expanded and then cropped when the insect body was at the edge of the image.

The datasets used in this study were made by simulating the Cifar10 dataset. Due to a shortage of field data, real-time image enhancement processing was performed while the data were used for training. That is, images were rotated randomly by 90 degrees, staggered by 0.5, and flipped up, down, left, and right.

#### ResNet V2 classifier

Kaiming He, the author of ResNet, proposed ResNet V2 in 2016 (He et al., 2016). On Cifar10, the author experimented with five residual unit structures using two networks, ResNet110 and ResNet164. The results with full pre-activation turned out to be the best. This may be due to that firstly; information transmission is unaffected by this structure; secondly, the batch normalization (BN) layer function acts as a pre-activation feature for regularization.

ResNet is a modular structure in which identical-shaped pieces can be stacked and connected (He et al., 2016). The residual unit was calculated as follows:


(1) $$y_{l}=h\,\left(x_{l}\right)+F\,\left(x_{l},W_{l}\right)$$

(2) $$x_{l+1}=f\,\left(y_{l}\right)$$

Following is the recursive calculation:


(3) $$x_{L}=x_{l}+\sum_{i=l}^{L-1}F\,\left(x_{i},W_{i}\right)$$

The link between any other L-th layer and any L-th layer is expressed in Equation (3). Assuming that the loss function is ε, the backpropagation formula is as follows:


(4) $$\frac{\partial \varepsilon }{\partial x_{l}}=\frac{\partial \varepsilon }{\partial x_{L}}\,\frac{\partial x_{L}}{\partial x_{l}}=\frac{\partial \varepsilon }{\partial x_{L}}\,\left(1+\frac{\partial }{\partial x_{l}}\,\sum_{i=l}^{L-1}F\,\left(x_{i},W_{i}\right)\right)$$

Followings are the three features of Equation (4):

(a) There are no impediments in transmitting gradient information between the two levels;

(b) $\frac{\partial \varepsilon }{\partial x_{l}}$, is not easily offset;

(c) When the weight is relatively small, the problem of gradient disappearance is successfully prevented.

Furthermore, the above formula’s properties are valid only if *h*(*x*_*l*_)=*x*_*l*_ and *x*_*l*1_=*y*_*l*_.

The ResNet V2 network has a total of 56 layers. The parameters for the training process of the ResNet V2 model are shown in Table 1, and the optimizer was Adam. Adam accelerated the convergence by using momentum and an adjustable learning rate. There were several advantages to this approach, including high computational efficiency, small memory requirement, and suitability for large parameters and non-fixed targets (Kingma and Ba, 2014). The loss function was based on the softmax layer’s cross-entropy function. Cross-entropy represents the distance between the actual output probability and the expected output probability; the smaller the value of cross-entropy, the closer the two probability. Notably, cross-entropy is currently the most widely used classification function in CNNs (Hui and Belkin, 2020).

**TABLE 1:** Parameters and resources used in training models.

Parameter or resource	Value or model
Batch size	16
Initial learning rate	1e-3
Optimizer	Adam
Epochs	200
Back end	TensorFlow 1.14
Python	3.6
Operation system	Windows 10
GPU	NVIDIA GeForce RTX 2060 Memory capacity: 6144 M Memory interface: 192 bit Memory Bandwidth: 336.1 GB/s
Categories	Helicoverpa armigera moth Pyralid moth Prodenialiture fabricius
The number of images used for training, validation, and test	6300, 1350, and 1350

#### Verification and comparison

##### Feature selection

For feature selection based on Dataset 1, the gradient boosting iterative decision tree (GBDT) method was applied. GBDT is a simplified serial integrated learning method that is different from other methods. The weak learner in GBDT must be a classification and regression tree (CART) model, and the loss of samples during model training must be as small as possible (Friedman, 2001). In this study, a total of 30 color, texture, and shape features were extracted for Dataset 1, Among them, the texture features were obtained by the statistics calculated by the gray co-occurrence matrix of four angles (0, 45, 90, and 135°), and the most relevant features were chosen for training and testing by GBDT.

##### Support vector machine and back-propagation neural network

Support vector machine and BPNN were used as baseline classifiers. The Gaussian kernel function was used in SVM, and the kernel function parameter gamma and penalty factor C were optimized using the particle swarm optimization (PSO) approach. The fitness value employed in the optimization process was the target identification rate obtained by cross-validation, and the optimal solution was found iteratively. The 10-fold cross-validation method was used for training after the optimized result was attained. In addition, BPNN employed 10-fold cross-validation for training, a Gaussian random distribution with a mean of 0 and a standard deviation of $1/\sqrt{{\text{n}}_{\text{in}}}$ to initialize the weights, the cross-entropy function as the cost function, and the L2 normalized cross-entropy to reduce overfitting. The stochastic gradient descent algorithm was used to train the input data; and simultaneously, the exponential learning rate decay was employed in training to ensure that the learning rate decreases with training to prevent both the delayed parameter updating caused by a low learning rate and the oscillation of parameters near the extreme value caused by a high learning rate. The number of input layer nodes was 11, the number of output layer nodes was 3, and the number of hidden layer nodes determined by an empirical formula (5) was 3.


(5) $$h=\sqrt{m+n}+a$$

Where *h* is the number of hidden layer nodes; *m*is the number of input layer nodes; *n* is the number of output layer nodes; and *a* is a constant between 1 and 10.

##### Evaluation criterion

The F1-score and the identification accuracy were used to assess the model’s performance. The proportion of all the model’s correct results to the total was used to evaluate the identification accuracy. The harmonic average F1-score of the precision and recall were used as a comprehensive metric to examine the model’s precision and recall. The greater the F1-score value, the higher the precision and recall. The calculation method for F1-score is as follows:


(6) $$F1-score_{i}=\frac{2*P_{i}*R_{i}}{P_{i}+R_{i}},\left(i=0,1,2,3\right)$$

(7) $$P_{i}=\frac{T\,P}{\left(T\,P+F\,P\right)}$$

(8) $$R_{i}=\frac{T\,P}{\left(T\,P+F\,N\right)}$$

Where P_i_ denotes the percentage of true i-type samples among the samples anticipated to be i-type; and R_i_represents the proportion of samples that are correctly predicted.

#### Methodology for updating models

The model must be updated as the number of insect species increases. To update the model, transfer learning and a hybrid dataset was applied in this study.

##### Model updating by using transfer learning

A new T_ResNet V2 model was created using the parameter transfer training of the ResNet V2 model based on the dataset collected in the field. Because of the uneven distribution of Dataset 2, 70 images from the fourth category were chosen at random and mixed with the other three categories to form Dataset 2-1 which was used in the transfer learning model. The initialization parameters of transfer learning were derived from the ResNet V2 model trained on Dataset 1. The parameters of the ResNet V2 model were changed during training, the fully connected layer parameters were eliminated, and Dataset 2-1 was used for training.

##### Model updating by using mixed dataset

A new dataset was created by filling or symmetric mixing of Datasets 1 and 2. Filling the mixing means with the first three categories of the training set of Dataset 2 were from Dataset 1 until the data quantity of the three categories of Dataset 2 was consistent with that of the fourth category. Symmetric mixing means that the data of the same category and the same quantity from the training sets of Dataset 1 and Dataset 2-1 were mixed, and the same quantity of images from the fourth category of Dataset 2-1 and the fourth category outside Dataset 2-1 were mixed. The validation set and test set of Dataset 2-1 were used for mixed data training, the model FM_ResNet V2 was obtained by filling-type mixed data training, and the model SM_ResNet V2 was obtained by symmetric mixed data training. Dataset 2-1 was used to compare the three models, and the test set was used to compare the three models’ performance.

Because the quantity of the data used to train transfer learning models was limited, the dataset was divided into the training set, a validation set, and test set in a ratio of 6:2:2. Real-time data enhancement including horizontal or, vertical flipping, randomly rotating (90 degrees), and random shear (0.5) to increase the data quantity during training.

### Identification and warning

The level of pest breakout was forecasted according to the number of pests. Following is the method for analyzing the number of pests in the images:

(a) The pest quantity study was conducted on a 7-day cycle since the emergence time of cotton bollworms and other pests is approximately 7 days.

(b) The increasing rate (IR) was used to classify the change trend of pest quantity in the first cycle.


$$I\,R=\frac{\left(\begin{array}{c} N\,u\,m\,b\,e\,r\,o\,f\,m\,o\,n\,t\,h\,s\,a\,t\,t\,h\,e\,m\,o\,m\,e\,n\,t\\ -C\,a\,r\,d\,i\,n\,a\,l\,n\,u\,m\,b\,e\,r\,o\,f\,m\,o\,t\,h\,s \end{array}\right)}{C\,a\,r\,d\,i\,n\,a\,l\,n\,u\,m\,b\,e\,r\,o\,f\,m\,o\,t\,h\,s}\times 100\%$$

(c) Since the second cycle, the day-on-day increasing rate (DIR) and the cycle-on-cycle increasing rate (CIR) were included for forecasting due to changes in environmental factors, such as temperature and humidity.


$$D\,i\,r=\frac{\left(\begin{array}{c} N\,u\,m\,b\,e\,r\,o\,f\,m\,o\,t\,h\,s\,o\,n\,t\,h\,e\,p\,r\,e\,s\,e\,n\,t\,d\,a\,y\\ -N\,u\,m\,b\,e\,r\,o\,f\,m\,o\,t\,h\,s\,o\,n\,t\,h\,e\,p\,r\,e\,v\,i\,o\,u\,s\,d\,a\,y \end{array}\right)}{N\,u\,m\,b\,e\,r\,o\,f\,m\,o\,t\,h\,s\,o\,n\,t\,h\,e\,p\,r\,e\,v\,i\,o\,u\,s\,d\,a\,y}\times 100\%$$

$$C\,I\,R=\frac{\left(\begin{array}{c} N\,u\,m\,b\,e\,r\,o\,f\,m\,o\,t\,h\,s\,o\,n\,t\,h\,e\,p\,r\,e\,s\,e\,n\,t\,d\,a\,y-N\,u\,m\,b\,e\,r\,o\,f\\ m\,o\,t\,h\,s\,o\,n\,t\,h\,e\,s\,a\,m\,e\,d\,a\,y\,i\,n\,t\,h\,e\,p\,r\,e\,v\,i\,o\,u\,s\,c\,y\,c\,l\,e \end{array}\right)}{\mathrm{Number}\,\mathrm{of}\,\mathrm{moths}\,\mathrm{on}\,\mathrm{the}\,\mathrm{same}\,\mathrm{day}\,\mathrm{in}\,\mathrm{the}\,\mathrm{previous}\,\mathrm{cycle}}\times 100\%$$

(d) Issuing warnings by the above analysis results.

The pest outbreak level was determined using the pest outbreak grading method for cotton field (Zhao et al., 2015). Pest incidence has five levels according to the technical criteria for pest survey (Table 2).

**TABLE 2:** Grading indexes of pest outbreak in cotton field.

Outbreak level	1	2	3	4	5
Pest density	≤5	>5∼10	>10∼20	>20∼30	>30
Pest increasing rate	0	>0∼100%	>100∼300%	>300∼500%	>300%

The pest increasing rate (PIR) was used to determine the pest outbreak level.


P⁢e⁢s⁢t⁢i⁢n⁢c⁢r⁢e⁢a⁢s⁢i⁢n⁢g⁢r⁢a⁢t⁢e=(N⁢u⁢m⁢b⁢e⁢r⁢o⁢f⁢n-l⁢e⁢v⁢e⁢l⁢p⁢e⁢s⁢t⁢s-N⁢u⁢m⁢b⁢e⁢r⁢o⁢f⁢l⁢e⁢v⁢e⁢l⁢ 1⁢p⁢e⁢s⁢t⁢s)N⁢u⁢m⁢b⁢e⁢r⁢o⁢f⁢l⁢e⁢v⁢e⁢l⁢ 1⁢p⁢e⁢s⁢t⁢s×100%

Where *n* = 1.., 5.

**FIGURE 6:**
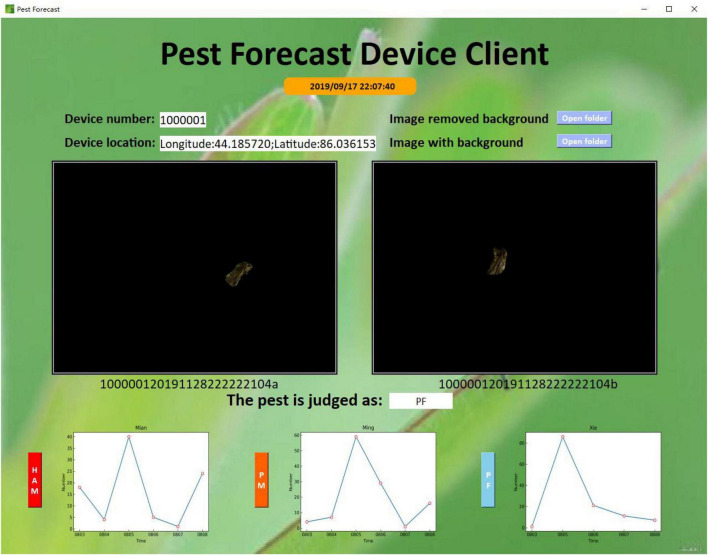
Program interface.

Finally, on the program interface (Figure 6), when any increasing rate (IR, DIR, or CIR) was in the range 0–100%, a blue warning was shown on the screen. When any increasing rate was between 100 and 300%, an orange warning was shown on the screen. When any increasing rate exceeded 300%, a red warning was shown on the screen.

## Result

### Analysis results of image acquisition feasibility

**FIGURE 7:**
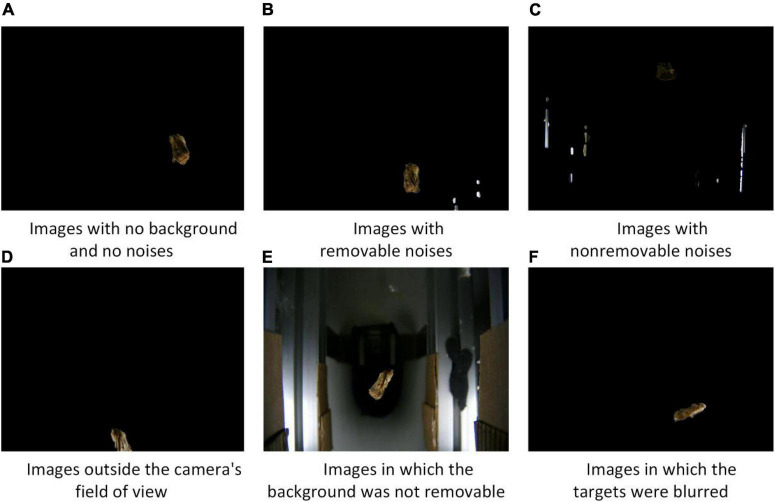
Image types.

All images (a, b, c, d, e, and f) were collected using the image acquisition device (Figure 7). The number of noise-free and noise-removable images accounted for more than 90.2 % of the images. Thus, the image acquisition device and method proposed in this study were viable.

### Feature selection results

Important features were extracted from the 30 features of Dataset 1 using GBDT’s method. The loss function was based on logarithmic loss, and the importance of each feature was calculated, with the mean value of the importance serving as the salient feature’s differentiating threshold. For the training and testing of SVM and BPNN, a total of 11 important features were used.

### Performance of the ResNet V2 model

**FIGURE 8:**
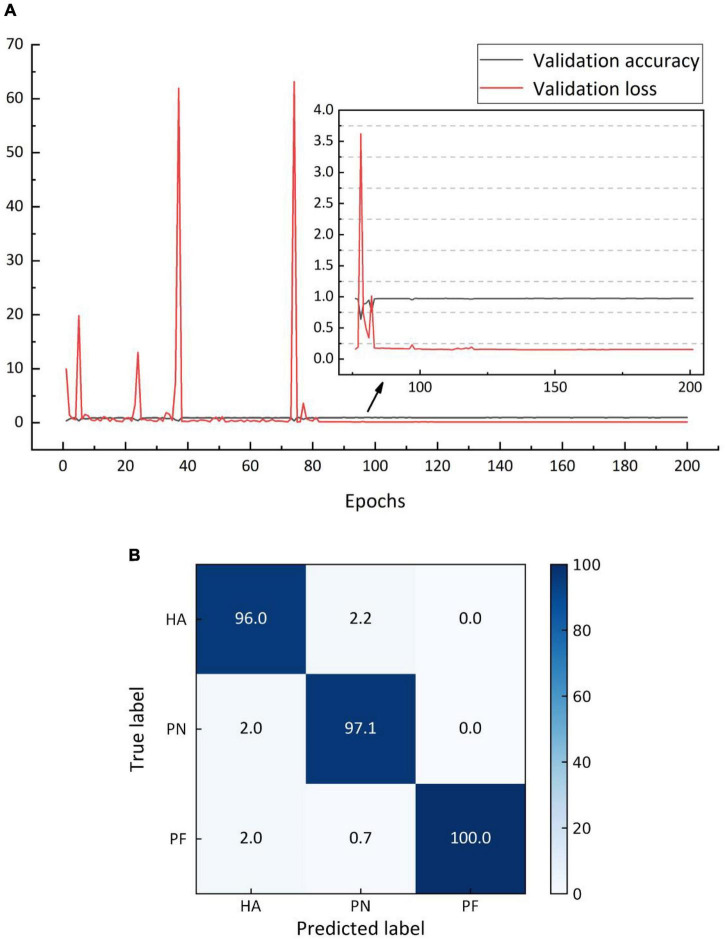
**(A)** Variation for identification accuracy and error based on validation set, **(B)** average classification accuracy by ResNet V2 (%).

Based on Dataset 1, ResNet V2 was trained for 200 cycles, with the validation set error calculated once in each cycle (Figure 8). The errors trended at approximately 0.15, with a minimum of 0.151, indicating that the model was not overfitted. Figure 8B shows the average identification accuracy based on the test set, with an identification accuracy of 97.7%. Table 3 shows the F1-score values for each category.

**TABLE 3:** Average identification accuracy and F1-score based on the test set.

Category	Accuracy (%)			F1-score (%)		
		
	SVM	BPNN	ResNet	SVM	BPNN	ResNet
*Helicoverpa armigera (HA)*	73.9	75.0	97.7	70.4	73.2	96.9
*Pyrausta nubilalis (PN)*				74.9	75.2	97.5
*Prodenialiture fabricius (PF)*				76.5	77.0	98.7

The optimal gamma and C values after 100 iterations of PSO SVM were 1.00 and 3.38, respectively, and the average identification accuracy based on the training and test sets were 74.3 and 73.9%, respectively. The average identification accuracy based on training set after 1050 iterations of BPNN was 76.4%, while that based on the test set was 75.0%. Table 3 shows the F1-score values produced by the SVM and BPNN for each category. Combining identification accuracy and F1-score value, it was found that the ResNet V2 model outperformed the others.

### ResNet V2, FM ResNet V2, and SM ResNet V2 model performance

**FIGURE 9:**
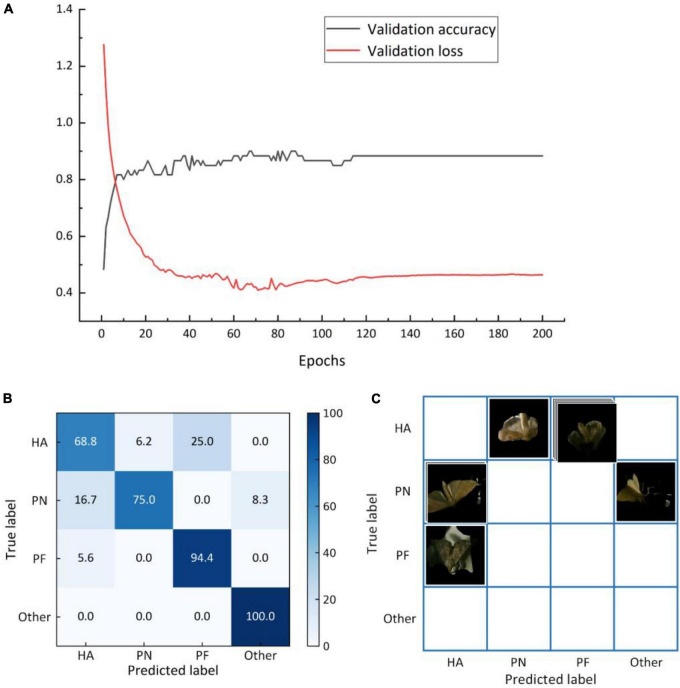
**(A)** Identification accuracy curve and error curve for the validation set(a), and matrix for the average identification accuracy based on the test set **(B)** and matrix for misclassified images **(C)** obtained by transfer learning.

A total of 200 training cycles were performed, and the error for the validation set was determined once per cycle; the best model was saved. Figure 9A depicts the variation of the curve for the verification set. The error was approximately 0.46, and the identification accuracy based on the validation set was 85.0%. The test set’s identification accuracy was 84.6% (Figures 9B,C and Table 4).

**TABLE 4:** The accuracy, training time, and F1-score of the three models.

Model	Accuracy (%)			Training duration(s)	F1-Score (%)	Label
	
	Training set	Validation set	Test set			
T_ResNet V2	93.5	85.0	84.6	586.16	47.3	0
					82.8	1
					86.1	2
					96.0	3
FM_ResNet V2	98.1	73.3	65.5	8189.08	25.8	0
					56.4	1
					88.3	2
					62.6	3
SM_ResNet V2	90.9	75.0	85.7	1041.82	50.0	0
					92.7	1
					82.1	2
					90.8	3

**FIGURE 10:**
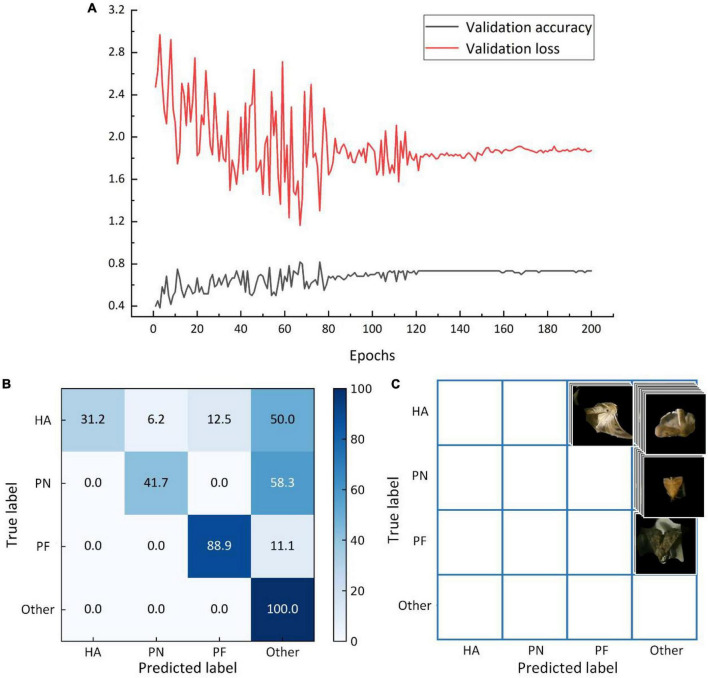
The average identification accuracy and error curves based on the validation set **(A)**, matrix of the average identification accuracy based on the test set **(B)**, and matrix of the misclassified images based on the test set **(C)** for the model trained on the filling mixed dataset.

**FIGURE 11:**
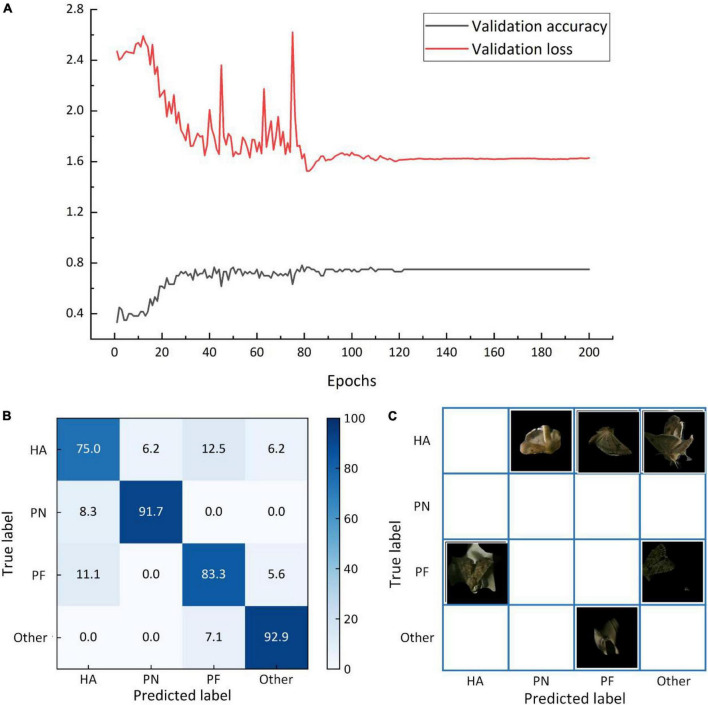
The average identification accuracy and error curves based on the validation set **(A)**, matrix of the average identification accuracy based on the test set **(B)**, and matrix of the misclassified images based on the test set **(C)** for the model trained on the symmetric mixed dataset.

The identification accuracy and error curves based on the validation set trained on the filling and mixed dataset are shown in Figure 10A. The identification accuracy was 73.3%, with an error of approximately 1.7. Based on the test set, the model trained based on the filling and mixed dataset had an identification accuracy of 65.5%. Figures 10B,C depicts the matrix, whereas Table 4 lists the F1-score values for each category. Figure 11A shows the identification accuracy and error curves based on the validation set trained on the symmetric mixed dataset, with an identification accuracy of 75.0% and an error of 1.6. The identification accuracy of the model trained on symmetric mixed dataset based on the test set was 85.7%. Figures 11B,C depicts the matrix.

## Discussion

### Background removal

The results of the image acquisition method’s feasibility demonstrates that using the Gaussian mixture model to remove the background in the image acquisition process is viable. The pest image acquisition in this study made use of the pest’s free fall and the sensor’s external triggering for image capture. The movement of the pest with relation to the background was used to remove the background. This can speed up the compilation of subsequent image datasets, and eliminate the need to tag pests in the image. However, this process is easily impacted by the environment, and it is difficult to completely remove the background. As a result, morphological methods with various parameter combinations should be used in conjunction with various settings to obtain the best possible background removal.

### Updating the classification model

The identification accuracy based on the verification and test sets was lower than that based on the training set, as shown by the training results of the filling mixed dataset (model FM_ResNet V2). This result may be because of the larger sample size of Dataset 1 of the filling mixed dataset than that of Dataset 2, which results in the validation set’s and test set’s internal variance is much lower than the training set’s. Therefore, the identification accuracy, recall rate, and precision based on the test set were lower. When the training results based on the symmetric mixed dataset and the transfer learning dataset (models SM_ResNet V2 and T_ResNet V2) were compared, the model SM_ResNet V2 was found to have higher identification accuracy than the model T_ResNet V2 based on the test set. Based on the verification set, however, the identification accuracy of the model SM_ResNet V2 was 10.7% lower than that based on the test set. This may be because the validation and test sets of the three models were all from Dataset 2-1 (see section “Verification and comparison”); among which, the model T_ResNet V2’s training set came from Dataset 2-1, and its data distribution was consistent with that of the validation set. However, the data distribution of the validation set was inconsistent with the training set for the model SM_ResNet V2. As a result, the accuracy based on the validation set was lower than that is based on the test set. Therefore, the model derived from the symmetrical mixed dataset was unable to effectively capture the distribution pattern of the new data in cotton field, resulting in lower identification accuracy. Furthermore, by examining the results of transfer learning, it was found that by combining existing model parameters with transfer learning, the model updating could be completed with a minimal amount of data, and the number of target categories could be altered as needed. The identification model is essential for the forecasting system. The updating of the model in a short time depending on the changes in the pest species which are beneficial for preventing outbreaks through real time monitoring, ensuring highly efficient and precise pest control.

### Early warning method

Traditional method for warning of pest outbreaks is based on field surveys. For example, five points are selected in a field to count the number of pest larvae on plants. In this study, pest images were used to predict the level of the pest outbreak. Combined with the traditional pest outbreak grading criterion, the increasing rates of pests were used to forecast the outbreak level. Early detection of pests during the moth stage is vital for preventing pest outbreaks. Although an early warning method for pest outbreaks was proposed in this study, multi-season verification tests are still needed to verify it further.

## Conclusion

This work demonstrated that the Resnet model has superior performance in classifying cotton field pests compared with SVM and BPNN models. This paper proposes a model update strategy based on the Resnet model. We found that the SM_Resnet V2 model obtained from the symmetric hybrid dataset performs better on the classification of new field data than the T_ResNet V2 model obtained from migration learning and the FM_ResNet V2 model obtained from the populated hybrid dataset. Moreover, the pest forecasting device proposed in this study can automatically collect and transmit the images of pests from cotton fields. The pest image acquisition method can acquire pest images by removing the background, and improving the identification accuracy. For target pests, the pest identification model based on ResNet V2 had a higher identification accuracy, and the application of transfer learning to update the identification model had excellent performance. The verification of the feasibility of the model updating by transfer learning, as well as pest identification and early warning methods, will be a focus for future research.

## Data availability statement

The original contributions presented in this study are included in the article/supplementary material, further inquiries can be directed to the corresponding author.

## Author contributions

CL and ZZ collected and analyzed the data and wrote the manuscript under the supervision of RZ and MZ. JB assisted in analyzing the data. All authors reviewed and revised the manuscript.
