# Corrigendum: Field pest monitoring and forecasting system for pest control

**DOI:** 10.3389/fpls.2022.1051704

**Published:** 2022-10-11

**Authors:** Chengkang Liu, Zhiqiang Zhai, Ruoyu Zhang, Jingya Bai, Mengyun Zhang

**Affiliations:** ^1^ College of Mechanical and Electrical Engineering, Shihezi University, Shihezi, China; ^2^ Key Laboratory of Northwest Agricultural Equipment, Ministry of Agriculture and Rural Affairs, Shihezi, China

**Keywords:** cotton pest, deep learning, image acquisition device, insect outbreak, transfer learning

In the published article, there was a mistake in the Correspondence email address for author Ruoyu Zhang. The email address was displayed as “zrjzju@gmail.com”. The correct email address is “zryzju@gmail.com’’.

The authors apologize for this error and state that this does not change the scientific conclusions of the article in any way. The original article has been updated.

## Publisher’s note

All claims expressed in this article are solely those of the authors and do not necessarily represent those of their affiliated organizations, or those of the publisher, the editors and the reviewers. Any product that may be evaluated in this article, or claim that may be made by its manufacturer, is not guaranteed or endorsed by the publisher.

